# Assessment of the understanding of informed consent including participants’ experiences, and generation of a supplemental consent decision aid for Gestational Diabetes Mellitus (GDM) research

**DOI:** 10.12688/hrbopenres.12811.1

**Published:** 2018-03-29

**Authors:** Shubham Atal, Fidelma Dunne

**Affiliations:** 1 Department of Pharmacology, All India Institute of Medical Sciences, Bhopal, 462022, India; 2 School of Medicine, Clinical Science Institute, National University of Ireland, Galway, Galway, SW4671G, Ireland

**Keywords:** Informed consent, Understanding, GDM, Pregnancy, Decision aid

## Abstract

**Background: **Informed consent is a basic ethical requirement of clinical research, yet deficiencies have been documented in the comprehension of its components among trial participants. Pregnancy research is sparsely conducted. Assessment of understanding of the informed consent among pregnant women suffering from Gestational Diabetes Mellitus enrolled in a randomized controlled trial, and their experiences was planned.

**Methodology: **A prospective observational cohort study was conducted among participants of EMERGE clinical trial at the University Hospital, Galway. Willing participants allowed observation of their consent encounters. They completed the standard QuIC questionnaire at follow up visits for assessment of objective and subjective understanding of informed consent, and reasons to participate and level of satisfaction. Data was entered and analysed using Microsoft Office Excel and Minitab version 18.

**Results: **The most commonly asked questions asked in the twenty consent encounters observed were focused upon the safety of the study drug for the developing foetuses and women. The general attitude of the women was positive towards participation. The mean objective understanding score was 72.43 ± 7 and the subjective understanding score was 91.67 ± 8.68 (out of 100). Critical components of consent like voluntarism, randomisation, withdrawal, and benefit to others were well understood. The domains related to nonstandard nature of treatment, additional risks/discomforts and compensation were poorly understood. The women cited the desire to provide benefit to future patients as the most common reason to participate, and most were satisfied with the consent process.

**Conclusion: **Comprehension of informed consent is good in most aspects, but the grasp of certain concepts is poor among the pregnant women. Efforts are needed to improve informed consent through engagement of investigators, research nurses and possibly, the use of a decision aid.

## Background

Informed consent is one of the pillars of ethically conducted clinical research. The first principle of the Nuremberg code states - *“The voluntary consent of the human subject is absolutely essential.”* ^1^ The International Council on Harmonisation of Technical Requirements for Registration of Pharmaceuticals for Human Use (ICH) good clinical practice guidelines (GCP) defines informed consent as “a process by which a subject voluntarily confirms his or her own willingness to participate in a particular trial after having been informed of all aspects of the trial that are relevant to the subject’s decision to participate.” ^2^ Informed consent embodies the principle of respect for persons and their autonomy which is one of the three main ethical principles set out in the Belmont report ^3^.

Certain ethical standards govern clinical research to ensure respect for human subjects, protection of their health and rights, and to minimize the possibility of exploitation ^4^. Informed consent is documented by means of a written, signed and dated informed consent form. The informed consent process is deemed to have the following requirements in order to be considered valid: voluntariness, capacity to make a decision, disclosure of information, comprehension, and finally the decision to participate ^5^. Patients need to understand the diagnosis, prognosis, nature and purpose of the intervention, alternatives, risks, and benefits. Participant satisfaction with the consent process is also a desirable outcome. The consenting physician or researcher should endeavour to convey the information to the fullest extent, both orally and/or in writing, in a manner and language which is appropriate and tailored to each individual’s level of understanding ^6^. However, it has been long argued that the informed consent process is often reduced to a simple recitation of the contents of the written document – participant information leaflet/sheet. As a result, there are numerous shortcomings which have been documented over the years regarding the participants’ understanding of consent, and what they are consenting to.

### Assessment of quality of informed consent

The quality of informed consent in clinical research is determined by the extent to which the research participants understand the process and different elements of informed consent ^7^. A number of studies have been done to assess the understanding or comprehension (i.e. quality) of informed consent. Systematic reviews have shown variations in the proportion of participants’ understanding of different components of informed consent. Tam *et al*. ^8^ reviewed 103 studies and showed that the components regarding purpose of the study, potential risks and adverse effects, confidentiality, availability of alternative treatments, and knowledge about comparability of treatments showed a relatively lower understanding among participants between 60 – 70 %. Mandava *et al.* ^9^ showed that contrary to the assumption, the understanding of different elements of informed consent varied considerably in both developing and developed countries, with little evidence of significant difference. Studies have revealed poor understanding of experimental and therapeutic aspects of clinical trials, and even lack of awareness about participation in research ^10^.

Self-completion questionnaires are a useful tool for assessment as they are shorter, easier to follow, cheaper and quicker to administer – an advantage in a clinical trial setting where participants are already burdened by the trial requirements on their visits ^11^. The Quality of Informed Consent (QuIC) is a brief, reliable, and valid questionnaire developed to assess the subject's grasp of important general concepts about clinical trials concepts followed largely from the U.S. Department of Health and Human Services’ basic elements of informed consent. It uses a scoring algorithm that avoids investigator bias ^10, 12^.

### Pregnancy research and improvement of informed consent

Traditionally, women have been considered a vulnerable population and excluded from clinical research. Due to the unique susceptibility of the maternal-foetal unit, pregnancy research has distinctive issues compared to normal clinical research ^13^. GDM is defined as “glucose intolerance with onset or first recognition during pregnancy.” ^14^ EMERGE *(A Randomised Placebo Controlled Trial of the effectiveness of Early MEtformin in Addition to Usual Care in the Reduction of Gestational Diabetes Mellitus Effects)* is a phase III randomized placebo controlled trial where pregnant women between 18 – 50 years of age diagnosed with gestational diabetes mellitus between 24 – 28 weeks of gestation are consented to participate in the study, and randomized to receive either different doses of the study drug metformin plus usual care or placebo plus usual care.

There are very few studies which focus on informed consent assessment exclusively in females or in clinical trials focused on conditions or diseases specific to females. In one such study ^15^, assessment of recall and understanding of the information given to the women about a barrier contraceptive showed that while most women had an understanding about participating in research, the comprehension regarding important aspects of the study and their participation varied among them. Some women were unsure about ease of withdrawal from the study. The risks associated with use of the contraceptive were understood by more than 80 % of the women but very few could actually understand the level of risk of pregnancy while using the experimental contraceptive. No studies have been published which measure the understanding of informed consent in a therapeutic intervention trial in pregnant women. In a recent study by Abay *et al*. ^16^, the effects of social, cultural, and religious factors during informed consent process on a proposed HPV-serotype prevalence study were assessed in healthy pregnant women. It was found that awareness about health research, rights of participants, and the condition being studied i.e. cervical cancer was low. An interesting finding was that most participants were sceptical and afraid of signing consent forms for research, while some also believed that taking part was their obligation.

Decision aids are known to improve patient treatment decisions; they appear to supplement and complement the informed consent literature ^17^. Juraskova *et al*. ^18^ reported successful piloting of a decision aid to assist women considering participation in a breast cancer prevention trial. The value of using qualitative research within or alongside randomised controlled trials (RCTs) is becoming more widely accepted. The term SWAT stands for ‘Study Within a Trial’; they can be conducted concurrently with the RCTs to improve any or all aspects of trial conduct ^19^.

### Rationale

The EMERGE trial gives us the unique opportunity to assess the quality of informed consent in the under-represented population of pregnant women in the setting of a randomized clinical trial. Previous studies on quality of informed consent have been carried out in trials of cancers, infectious diseases, cardiovascular diseases, neurological disorders, vaccines, surgical interventions, paediatric conditions, and other miscellaneous conditions. Pregnant women and GDM research have not been included in such studies. GDM is a niche area of research focusing on a significant problem specific to pregnancy. The participation of women in such research is likely to present a different or unique set of challenges.

This study also provided a chance to assess any concerns regarding the protection of research participants at a clinical research site like this one in Ireland where a variety of clinical trials are being conducted. A ‘consent decision aid’ was planned to be developed using the findings of the study to aid future trial participation.

## Methods

The study was carried out with the primary objective of evaluating whether participants in a GDM research based Randomized Controlled Trial (EMERGE), objectively and subjectively comprehend the basic elements of the informed consent that they have provided, and to identify the domains which are at risk of being misunderstood. The secondary objectives were to find the common questions/concerns raised by the participants with the aim of designing a consent decision aid for future participants in the trial, and the factors which motivate the participants to enrol in GDM research, as well as to assess the overall attitude and level of satisfaction of the participants regarding participation.

## Ethics and consent

The study was approved by the Clinical Research Ethics Committee, Galway University Hospitals; Ref. C.A. 1726 vide letter dated 4th April 2017. Written informed consent was gathered from all participants completing the study questionnaire.

**Study design:** The study was carried out as an observational cohort study with prospective data collection over a period of 4 months.

**Study site:** The study was conducted at the HRB Clinical research facility, Galway, and the Galway University Hospital, Galway, Ireland.

**Study participants:** Consecutive patients who engaged in discussions to consider consenting to participate in the EMERGE trial during the study period were invited to participate in this study.

**Eligibility criteria:** All participants engaging in consent discussions with the investigators regarding participation in the EMERGE clinical trial were considered eligible. Participants not giving verbal and/or written consent to participate in the study were excluded.

### Data Collection

The direct observation of consent meeting was done by the authors of this study after seeking verbal consent from potential EMERGE participants at the beginning of their meeting with the trial investigator. Subsequently, the study questionnaire was completed by the willing participants (who had enrolled in EMERGE) at one of the first two follow up visits at 2 weeks or 4 weeks post screening, after getting written informed consent from them.

***Study questionnaire***. A validated standardized questionnaire - the Quality of Informed Consent (QuIC), was suitably modified and adapted for the study. ( Supplementary File 1) The QuIC is a simple reliable instrument for assessment of informed consent process in clinical trials. It has 2 parts – A and B, aimed at eliciting participants’ responses regarding their objective and subjective understanding respectively of the various aspects of the informed consent process; an indicator of the quality and adequacy of the consent process they underwent and their experiences towards it ^10^.

At the end of the questionnaire, two additional standard questions taken from similar questionnaires regarding factors that motivate patients to participate in clinical research and their overall satisfaction with the consent process were added. Demographic details like age, parity, race, and level of education were collected as part of the questionnaire to characterise the participants in this study.

***Questions/concerns raised by the participants***. The common questions or concerns raised by the participants during their discussion/s with the trial physicians and research nurses of the EMERGE trial in the consent meeting/s were observed by the study investigator unobtrusively and recorded in the form of participant notes – an open ended direct observation. These observations were then immediately corroborated with both the consenting investigator and the research nurse involved in the consent discussion/s, to ensure no concerns were missed, and to validate the observations made by the study investigator. These questions or concerns were used as the basis to develop a simple supplemental ‘consent decision aid’ to help in better decision making for future patients ^20^.

Additionally, the verbatim remarks/comments of the participants related to their participation in the trial, their overall behaviour or any other remarkable aspect of their interaction during the consent meeting were carefully noted to assess their general attitude towards participating in clinical research. These observations were then immediately discussed briefly and corroborated with investigator and/or the study nurse.

### Written consent for participation in the study

Verbal consent was taken from potential participants of the EMERGE trial for allowing observation of their consent meeting/s with the trial investigators to collect data on commonly raised questions / concerns and participants’ attitudes. Written informed consent was subsequently taken for assessment of quality of consent by administration of the study questionnaire from the women who had consented to participate in the EMERGE trial participants. They were provided with the participant information sheet and consent form version 3.0. dated 29/03/2017 for this study ( Supplementary File 2) at the end of their consent meeting for EMERGE.

### Sample size

With an expected recruitment rate of 15 participants per month (60 in 4 months) for the EMERGE trial and an expected recruitment of 50 – 70% of these participants in our study based on previous similar studies ^21, 22^ we expected 30 subjects to complete the study questionnaire in the study duration of 4 months. However, due to delay in the start of the recruitment in the trial and due to slow recruitment rate initially, a sample size of 15 participants could be attained, which had a margin of error of 0.775 for the QuIC score results based on an expected Standard Deviation of 1.4 for the mean summary scores obtained ^21, 22^.

### Statistical analysis

Simple descriptive statistics were used to display and analyse data using frequencies, percentages, range, means/medians, and standard deviations. Study questionnaire responses were evaluated and analysed using the scoring algorithm available for the questionnaire. In part A (objective understanding) of the modified QuIC, correct answers for each question were assigned a score of 100 points, incorrect answers were assigned a score of 0 points, and “unsure” response was assigned a score of 50 points. Resulting summary score potentially ranged from 0 to 100 obtained by averaging the domain wise scores for all completed questions in each of the 13 designated domain ^10^. For part B (subjective understanding) of the QuIC, the responses to each of the 14 questions were averaged. The raw average (range 1–5) was then scaled from 0–100 as follows: summary score = (raw average − 1) × 25. Microsoft Excel 2016 and Minitab version 18 software were used for the statistical analysis.

## Results

During the four months between May to August 2017, twenty pregnant women diagnosed with GDM were involved in consent discussions with the investigators of the EMERGE clinical trial as potential participants at the CRF and University hospital Galway. Observations were made on these participants to collect data. The results are described below in the order in which data was collected from each participant.

### Consent meetings of the participants with the trial investigators

Twenty consent meetings between the potential participants and trial investigators were directly observed after seeking verbal consent from the women. Seventeen of these women consented to enrol in the trial among which one participant each was lost due to screen failure and withdrawal respectively.

Table 1 gives a description of some of the important parameters of the 20 consent meetings. A total of 4 investigators – one principal investigator and three sub investigators, conducted these meetings. Out of the 17 participants who gave consent to enrol in the trial, 14 did so in their first meeting with one of the trial investigators and the other 3 needed two meetings to provide consent. The number of questions or concerns raised by the participants ranged from none to 6 questions. Most commonly, 2 or 3 questions were asked actively by the participants. The duration of the consent meetings ranged from 15 minutes to 90 minutes, the average duration being 32 minutes.

**Table 1.** Description of informed consent meetings with investigators.

Characteristics of the consent meetings		No. of participants
No. of potential participants observed	20	Consented: 17 Not consented: 3 Consent withdrawn:1 Screen failure: 1
No. of consenting investigators	4	Investigator 1 (PI): 11 Investigator 2: 3 Investigator 3: 5 Investigator 4: 1
No. of consent meetings in which Research Nurse (RN) was present	18	RN 1: 14 RN 2: 4
No. of meetings required for giving informed consent	(17 consents)	Consented in first meeting: 14 Consented in second meeting: 3
No. of questions asked by participants in consent meetings		0 question : 4 1 question : 2 2 questions : 5 3 questions : 4 4 questions : 2 5 questions: 2 6 questions: 1
Duration of consent meetings	(20 meetings)	Average duration : 32 mins. Maximum duration : 90 mins. Minimum duration: 15 mins.
Accompanying person/s in consent meetings	(20 participants)	None: 13 Spouse/partner: 5 Mother: 1 Friend: 1

### Participants’ demographics

All the 17 participants who consented for the EMERGE clinical trial gave written informed consent to further participate in this study by filling out the study questionnaire at one of their first 2 follow up visits for the trial, thus giving a 100 % enrolment rate. The modified QuIC questionnaire was eventually administered to 15 participants who came for their follow up visits; one participant each was lost due to screen failure and withdrawal respectively. Table 2 shows the demographic characteristics of the participants.

**Table 2.** Demographic characteristics of participants.

Characteristic	Participants (N = 15)
Age (mean ± SD) in years	32.40 ± 4.08 Min.: 24 Max.: 41
Ethnicity	Irish: 10 Polish: 2 French: 1 Euroasian: 1 Croatian: 1
Education	Leaving certificate: 5 Bachelor’s degree: 8 Master’s degree: 2
Parity	Nullipara: 9 Primipara: 2 Multipara: 4

### Questions / concerns raised by participants

During their consent meetings, the participants raised several questions or concerns related to the trial. These questions were noted down, and a list of the most frequently asked questions along with their answers was compiled for as a ‘consent decision aid’ for potential future participants to help allay the common concerns and queries. ( Supplementary File 3) The concern most commonly raised by the participants was regarding the safety of the study drug metformin for their babies (12 out of 20 participants). The other common concerns raised were also related to the study drug – its side effects for the women (8 participants), previous evidence of its use in pregnant women or GDM (7 participants), and its potential benefits (6 participants). Some participants also wanted to know how many women would be participating in the trial, how many have enrolled till now and the time required to be spent for the study in terms of number of visits and duration of the visits.

### General attitude of the participants

Generally, it was seen that the women consenting to enrol in the trial were positive, confident, and genuinely willing to participate in a clinical research study. Some of them did have apprehensions regarding taking part in the trial, particularly due to safety concerns for their developing foetus, but most women were relaxed and receptive. There was expression of disappointment with the diagnosis of GDM in some of the participants which was expected. It was interesting to note that some participants also seemed to specifically harbour hopes of receiving the active drug i.e. metformin.

Some of the interesting remarks are as follows:

*"Someone has to take initiative to participate in these studies."*

*"Feel like a guinea pig"* (expressed light heartedly)

*"Someone should participate in research studies."*

*"Happy to do something that may help in the future."*

*“Hope the treatment works.”*

*“Not entirely sure if she should start taking medications right away.”* (spouse’s comment)

*“Wish I get placebo.”*

*“I understand the basics of the study well, read about the uses of metformin.”*

*“I am happy to enrol.”*

*“Shocking that metformin is not yet widely used in Ireland for GDM.” “Feel good to be in the study, I shouldn't be panicking.”*

*“There is a lot of signing to do.”*

*"I have faith in my consultant - if he is fine with me being in the study then I am confident." “It’s important to know if the drug has been used in other countries before because I wouldn't want to be a total guinea pig.”* (Disappointed upon being informed of being a screen failure)

*“I am hopeful that I get metformin, I want to avoid taking insulin later.”*

*“Want to avoid taking insulin.”*

*“Had 6 children previously but never had a sugar problem.”*

*“My family was sceptical - why do you want to do something like this, but I was confident I wanted to do this.”*

### QuIC questionnaire Part A: Objective understanding of participants

This part of the questionnaire evaluates the objective understanding of informed consent among the participants in 13 independent domains, spread across 16 questions. There were three responses available to the questions in section A: (1) agree, (2) unsure, and (3) disagree.

Table 3 shows the proportion of the three responses given by the participants for each of the questions.

**Table 3.** Objective understanding of informed consent among participants (QuIC Part A).

Questions	Participants responses (N = 15)		
	Agree n (%)	Unsure n (%)	Disagree n (%)
A1. When I signed the consent form for my current therapy, knew that I was going to participate in a clinical trial.	15 * (100)	0 (0)	0 (0)
A2. The main reason clinical trials are done is to improve the treatment of future patients.	15 * (100)	0 (0)	0 (0)
A3. I have been informed how long my participation in the clinical trial is likely to last.	15 * (100)	0 (0)	0 (0)
A4. All the treatments and procedures in my clinical trial are standard for my type of condition.	**13** **(86.67)**	2 (13.33)	0 * (0)
A5. In my clinical trial, one of the researchers’ main purposes is to compare the effects (good or bad) of two or more different ways of treating patients with my type of condition, in order to see which is better.	14 * (93.33)	1 (6.67)	0 (0)
A6. The treatment being researched in my clinical trial has been proven to be best for my type of condition.	**13** **(86.67)**	2 (13.33)	0 * (0)
A7. After I agreed to participate in my clinical trial, my treatment was chosen randomly (by chance) from two or more possibilities.	14 * (93.33)	1 (6.67)	0 (0)
A8. Compared with standard treatments for my type of condition, my clinical trial does not carry any additional risks or discomforts.	**11** **(73.33)**	1 (6.67)	2 * (13.33)
A9. There may not be direct medical benefit to me from my participation in this clinical trial.	15 * (100)	0 (0)	0 (0)
A10. By participating in this clinical trial, I am helping researchers learn information that may benefit future patients.	15 * (100)	0 (0)	0 (0)
A11. Because I am participating in a clinical trial, it is possible that the study sponsor, various government agencies, or others who are not directly involved in any care could review my medical records.	8 * (53.33)	3 (20)	4 (26.67)
A12. My doctors did not offer me any alternatives besides treatment in this clinical trial.	**5** **(33.33)**	2 (13.33)	8 * (53.33)
A13. The consent form I signed describes who will pay for treatment if I am injured or become ill as a result of participation in this trial.	5 * (33.33)	**8** **(53.33)**	2 (13.33)
A14. The consent form I signed lists the name of the person (or persons) whom I should contact if I have any questions or concerns about this clinical trial.	14 * (93.33)	1 (6.67)	0 (0)
A15. If I had not wanted to participate in this clinical trial, I could have declined to sign the consent form.	15 * (100)	0 (0)	0 (0)
A16. I will have to remain in the clinical trial even if I decide someday that I want to withdraw.	2 (13.33)	0 (0)	13 * (86.67)

*Correct answer

Correct answers were obtained for many important domains of informed consent. All participants (100 %) correctly answered the statements on consent for research participation (A1), expected duration of participation (A3), no direct benefit (A9), possible benefit to others (A10), and voluntary nature of participation (A15). More than 90 % gave correct answers for statements on explanation of the purpose of research (A2, A5), concept of randomisation (A7), and the explanation of whom to contact in case of questions or emergencies (A14).

More than 70 % of participants incorrectly answered statements about identification of procedures that were experimental (A4), the study treatment being proven the best (A6), and a description of foreseeable risks or discomforts (A8). About half of participants could correctly answer statements on disclosure of alternative procedures or courses available (A12) and only one third understood the explanation of procedures for compensation in case of trial related illness or injury (A13), for which half the participants were unsure.

### QuIC questionnaire Part B: Subjective understanding of participants

Part B of the QuIC questionnaire contained 14 questions. These questions asked the participants to rate how well they felt they understood each of the domains of informed consent. Table 4 shows the proportion of the responses given by the participants for each of the questions on a Likert scale of 1 – 5; where 1 stands for ‘I did not understand this at all’ and 5 stands for ‘I understood this very well’.

**Table 4.** Subjective understanding of informed consent (QuIC Part B).

Questions	Participants responses (N = 15)					
	1 n (%)	2 n (%)	3 n (%)	4 n (%)	5 n (%)	Mean response ± SD
B1. The fact that your treatment involves research	0 (0)	0 (0)	0 (0)	2 (13.33)	13 (86.67)	4.87 ± 0.35
B2. What the researchers are trying to find out in the clinical trial	0 (0)	0 (0)	0 (0)	3 (20)	12 (80)	4.8 ± 0.41
B3. How long you will be in the clinical trial	0 (0)	0 (0)	0 (0)	2 (13.33)	13 (86.67)	4.87 ± 0.35
B4. The treatments and procedures you will undergo	0 (0)	0 (0)	0 (0)	1 (6.67)	14 (93.33)	4.93 ± 0.26
B5. Which of these treatments and procedures are experimental	0 (0)	0 (0)	1 (6.67)	1 (6.67)	13 (86.67)	4.8 ± 0.56
B6. The possible risks and discomforts of participating in the clinical trial	1 (6.67)	0 (0)	1 (6.67)	3 (20)	10 (66.67)	**4.4 ± 1.12**
B7. The possible benefits to you of participating in the clinical trial	0 (0)	0 (0)	1 (6.67)	1 (6.67)	13 (86.67)	4.8 ± 0.56
B8. How your participation in this clinical trial may benefit future patients	0 (0)	0 (0)	0 (0)	1 (6.67)	14 (93.33)	4.93 ± 0.26
B9. The alternatives to participation in the clinical trial	0 (0)	0 (0)	1 (6.67)	1 (6.67)	13 (86.67)	4.8 ± 0.56
B10. The effect of the clinical trial on the confidentiality of your medical records	0 (0)	0 (0)	2 (13.33)	2 (13.33)	11 (73.33)	4.6 ± 0.74
B11. Who will pay for treatment if you are injured or become ill because of participation in this clinical trial	3 (20)	1 (0)	6 (40)	0 (0)	5 (33.33)	**3.2 ± 1.52**
B12. Whom you should contact if you have questions or concerns about the clinical trial	1 (6.67)	0 (0)	0 (0)	1 (6.67)	13 (86.67)	4.67 ± 1.05
B13. The fact that participation in the clinical trial is voluntary	0 (0)	0 (0)	0 (0)	1 (6.67)	14 (93.33)	4.93 ± 0.26
B14. Overall, how well did you understand your clinical trial when you signed the consent form	0 (0)	0 (0)	0 (0)	4 (26.67)	11 (73.33)	4.73 ± 0.46

*(Likert scale responses 1 – 5; 1 = Did not understand at all, 5 = Understood very well)*

**SD = Standard Deviation**

Participants felt most comfortable with their understanding (4.93) about treatments and procedures involved (B4), expectation of benefit to future patients (B8), and voluntary nature of participation (B13) followed by their understanding (4.87) about the treatment involving research (B1) and duration in the trial (B3). The understanding was also quite good (4.8) for statements B2, B5, B7, and B9 which are concerned with purpose of research, experimental nature of research, possible benefits, and alternatives available respectively.

Participants felt least comfortable with their understanding of the procedures and compensation in case of study-related injury or illness (B11); the mean response being 3.2 ± 1.52 with three participants registering 1 as their response which means not understood at all. Participants were also relatively less comfortable with their understanding (4.4) of the possible risks and discomforts of participating in the clinical trial (B6).

It was interesting to note that some domains which had low proportion of correct understanding in the objective evaluation in part A of questionnaire, were perceived to be understood well to very well by participants.

### Scoring of the participants’ responses on the QuIC questionnaire

Table 5 shows the summary knowledge scores in part A and the summary self assessment scores in part B of the questionnaire, obtained by the participants along with the mean summary scores. 

**Table 5.** Summary scores of participants’ understanding of consent on the QuIC questionnaire.

Type of understanding	Summary score (0 – 100)															
	P 1	P 2	P 3	P 4	P 5	P 6	P 7	P 8	P 9	P 10	P 11	P 12	P 13	P 14	P 15	Mean ± SD, *(95% CI)*
**Objective** **(QuIC A)**	65.38	65.38	75	82.69	76.92	73.08	69.23	69.23	75	69.23	69.23	88.46	69.23	61.53	76.92	**72.43** **± 7** **(68.83, 77.64)**
**Subjective** **(QuIC B)**	91.07	92.75	89.25	92.75	96.5	100	78.5	100	96.5	96.5	69.75	100	96.5	92.75	82.25	**91.67** **± 8.68** **(86.09, 97.84)**
Duration of consent meeting (mins.)	25	20	25	45	30	35	90	20	30	35	20	25	30	30	25	**32.33** **± 17.31**
No. of days from consent to completing QuIC	15	14	15	18	22	22	26	14	20	28	20	13	14	14	15	**18** **± 4.78**

*P = Participant, Scoring range: 0 – 100 (0 – lowest understanding, 100 – highest understanding)* *SD = Standard deviation*

On an average, the participants completed the study questionnaire 18 days from the day of providing informed consent for the EMERGE trial.

The average summary knowledge score on QuIC Part A obtained by the participants was 72.43 (± 7) out of a possible 100 which is the highest understanding. The summary scores ranged between 61.53 and 88.46. Since there is no cut off or a standard summary score suggested for good understanding, it is assumed that the closer the score is to 100, the better the understanding is. The average self-assessment score obtained by the participants on QuIC Part B was quite higher at 91.67 (± 8.68). Three of the participants perceived their understanding to be the highest possible, obtaining a perfect self-assessment score of 100.

**Figure 1.**
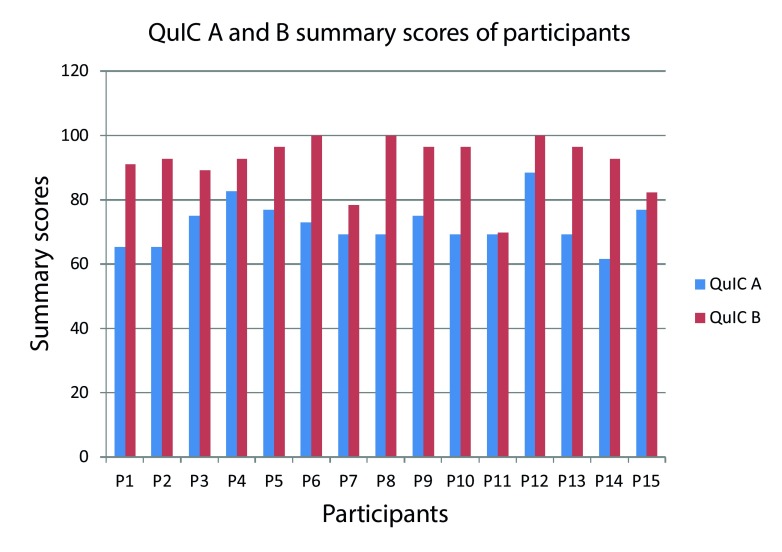
QuIC Part A and Part B summary scores of the participants.

It can be seen from Figure 1 that the self-assessment summary scores (QuIC B) are consistently higher than the knowledge summary scores (QuIC A) in all the participants. This shows that in general, the participants *felt* they understood the different components of informed consent better than they actually understand them. Upon trying to establish a correlation between these objective and subjective summary scores of the participants, it was found that there was a very weak linear relationship as demonstrated by the Pearson correlation coefficient *r* of + 0.206. This correlation was not significant though as the p value was 0.461.

### Reasons for participation and overall satisfaction with consent process

Table 6 shows the reasons attributed by the trial participants for their participation in the trial, as well as their overall level of satisfaction with informed consent process of the EMERGE trial as indicated by them on the 5-point Likert scale styled responses from Very satisfactory to Very unsatisfactory.

**Table 6.** Reasons for participating and level of satisfaction of participants.

Reasons or motivation to participate in the trial	No. of responses (N = 15) n (%)
A. I thought the trial offered the best treatment available	9 (60)
B. I believe results from the trial could benefit other patients in the future	14 (93.33)
C. I want to contribute to scientific research	12 (80)
D. I believe I will be monitored more closely as part of this trial	10 (66.67)
E. I believe the quality of care I receive will be better as part of this trial	8 (53.33)
F. My family were keen for me to participate	2 (13.33)
G. I trusted the doctor treating me	7 (46.67)
H. I think my condition will get worse unless I take part in the trial	1 (6.67)
I. Other reasons	0 (0)
Participants’ level of satisfaction with informed consent process	
Very satisfactory	12 (80)
Satisfactory	0 (0)
Neutral	1 (6.67)
Unsatisfactory	1 (6.67)
Very unsatisfactory	1 (6.67)

The table shows that the most common reasons that the participants cited as their motivation to take part in the trial were: the belief that the trial could benefit other patients in the future (93.33 %), contribution to scientific research (80 %), and benefit of better monitoring with trial participation (66.67 %). Most of the participants (80 %) were very satisfied with the informed consent process they underwent for enrolment in the trial.

## Discussion

The study shows that while most participants exhibited a good overall understanding of key elements of informed consent, they did not comprehend some of the elements. There was a poor grasp of the experimental nature of therapy, foreseeable risks or discomforts, and compensatory procedures. However, the perceived understanding of the participants was very good for all the elements of informed consent. These findings are in agreement with findings of similar prior studies conducted on different subsets of research participants and extend prior knowledge of issues identified in consent understanding in clinical research. More importantly, the study specifically documents such issues among pregnant women in clinical research and in GDM research.

Most participants were found to be concerned about the safety of the study drug for their foetuses. Generally, the attitude of the women towards participation in the clinical trial was positive. Majority of the participants were ‘very satisfied’ with the informed consent process for the trial. The most common reason to enrol in the research was the belief that the trial could benefit other patients in the future.

### Objective understanding of informed consent

The mean QuIC-A summary score obtained in the study, from a maximum possible score of 100, was 73.24 ± 6.93 which is only slightly lower than the one reported (77.8 ± 9.4) in the original cross-sectional validation study of QuIC by Joffe *et al*., ^22^ in cancer trial participants. Other similar studies have reported the mean knowledge score to range from 69.3 to 77.6.75 ^23, 24^. Thus, in the setting of RCTs using the QuIC, the study results compare favourably and are similar overall. The systematic reviews ^8, 9^ conducted on consent assessment studies have demonstrated that certain elements of informed consent are almost universally difficult to grasp for participants. Our study demonstrated a similar pattern and major deficiencies were observed in understanding the nonstandard and experimental nature of the study treatment, the risks or discomforts related to the study treatment, the disclosure of alternative treatment available, and the procedure for compensation in case of trial related injury/illness.

***Understanding study risks including confidentiality and compensation***. Results showed that 75 % of participants thought that the research does not carry additional risks or discomforts. This is in congruence with earlier findings that trial participants do not realise that the research might carry incremental risk. The studies by Kilma J *et al*. ^21^ and Ormond *et al*. ^23^ in parents consenting for a biobanking study in their children showed that 87.3 % and 81% of responses were wrong respectively, whereas a recent study among cancer trial patients reported this proportion as 54 % ^25^. In the context of this RCT, it is noteworthy that the study drug although being tried in Ireland, as an initiation therapy, for the first time, is actually accepted and used as a standard first line treatment in some other leading Western countries ^26– 28^. This knowledge, obtained from the study PIL and consent discussions, may have led the participants to be assured that the study treatment *would not have additional risks*; the expected risks were presumed to be standard for the treatment.

These findings are consistent with previous studies where respondents minimized the risks of research participation ^29, 30^. The participants failed to recognize the nonstandard nature of the use of study drug, and they consented after feeling satisfied with the evidence presented to them regarding safety of the drug for their developing babies. Therefore, it is possible that they deemed the study to be relatively risk free. Participants have been known to have shown inability to recognize minor study related risks, and to presume treatments to be standard as they trusted the doctor or institution conducting the study ^23^.

The concept of confidentiality of records identifying the subjects was relatively less understood in both objective (58.33 %) and subjective assessments; similar to the results reported by previous studies which range from 50 % to 66.2 % ^21, 22, 23^. It can be hypothesized that this might be due to comparatively less emphasis in explaining on the part of the investigators, and less interest in knowing on the part of the participants regarding confidentiality as compared to the safety and efficacy issues. More than half the participants were also unsure of who would pay for injuries/illness related to the trial. Again, this corresponds to the results obtained in previous studies which have shown 30 – 70 % of respondents being wrong or unsure about the question ^21, 23, 31^.

***Understanding nature of treatment, potential benefits: therapeutic misconceptions?*** All the participants in the study correctly understood that they may not receive direct benefit and that the study may benefit future patients. However, most women incorrectly thought that the study treatment in the trial was standard and proven as best for GDM. It has been suggested that participants of a RCT, particularly a phase 3 trial, enrolled in the standard group may misinterpret these questions to refer to their own group rather than to the trial as a whole ^22^. The standard treatment arm in EMERGE consists of the usual care in the form of dietary and exercise interventions given at the time of diagnosis of GDM (along with placebo). The study drug has been proven to be safe and effective for treating GDM in previous studies in other countries, Therefore, it could be hypothesized that the participants deemed the study drug to be standard and already proven as best and misinterpreting the fact that it is in fact an experimental therapy in Ireland when initiated at time of diagnosis.

Another reason could be ‘therapeutic misconception’; participants agreeing to take part in a clinical trial seem to expect substantial benefit from the experimental study treatment, considering it to be novel and therefore better ^32^. Furthermore, information that contradicts the therapeutic misconception might be difficult for patients to assimilate in the context of their natural hopes and anxieties ^33^. A few other reasons have been postulated as to why participants in clinical trials might fail to correctly comprehend the information conveyed to them like not understanding the way information was conveyed, forgetting the information over time, or not understanding the questions during evaluation ^15^.

***Understanding randomization, voluntarism, and withdrawal***. In our study, 100 % of participants had the correct understanding of the concept of random allocation of treatment and also *felt* they understood it very well. While the voluntary nature of participation and opportunity to withdraw have been shown to be understood well in previous studies and systematic reviews, the concept of randomization has been identified as one of the trickier concepts, understood in only half the studies in one review ^32^ and half of the participants in another review ^8^. Similarly, a much higher proportion of women in the study displayed objective and subjective understanding of voluntarism (100 %) and right to withdrawal (83.33 %) compared to the combined results in systematic reviews.

### Subjective understanding of informed consent

It was seen that the participants’ rating for perceived understanding of most of the components of consent was high to very high; a mean understanding of > 4.5 for knowledge of involvement in research, procedures in the trial, experimental and nonstandard nature of treatment, benefits from research, alternatives available, confidentiality, and voluntary nature of participation. The only aspects where participants were unsure to some extent were related to the procedures of compensation, the risks / discomforts, and contact persons for the trial. The mean subjective self-assessment score obtained in the study was 91.96 ± 9.24. This compares with the subjective score of 91.5 obtained in the study by Jefford *et al*. ^24^ and score of 89.6 obtained in the study by Ormond *et al*. ^23^ This high summary score shows that the subjective understanding was very high for even those components for informed consent which were found deficient in the objective assessment (previous section). Possibly, this could be because the participants *felt* they understood well what they deemed was correct although it was technically incorrect.

### Concordance between actual and perceived understanding

There was a high degree of concordance in the two types of understanding for most of the components of informed consent. But the concordance was relatively low for the alternatives available and confidentiality, whereas there was a discrepancy in responses obtained for the experimental nature of therapy and risks / discomforts of the study. A comparison of the summary knowledge (QuIC A) and self-assessment (QuIC B) scores shows the objective understanding scores to be consistently higher than the subjective understanding scores in all the 15 participants. It is apparent that research participants appear to think that they understand more than they actually do.

### Correlation of understanding with duration of consent meetings and time since signing consent

The systematic review by Tam *et al*. ^8^ has shown that there is an association between the understanding of certain components of informed consent and the time elapsed since giving the consent – the closer the assessment is to the time of giving consent, the better the understanding. But such an association could not be found in this study. No association was found between these factors and the QuIC scores in the original study with QuIC by Joffe *et al*. ^22^ and the latest study conducted by Schumacher *et al.* as well ^25^. In our study, the small sample size was probably not sufficient to demonstrate such an association, if it was present. Moreover, the consent process in EMERGE included preliminary discussions with the research nurses, so the participants may not have needed lengthy discussions with the investigators.

### The consent meetings – analysis of observations

Direct observation of the actual informed consent process allows better understanding of the context of the encounter. It was observed that almost half of the consent discussions between participants and investigators lasted half an hour or more. There is no documentation of consent duration in similar settings of pregnancy research. In the Joffe study ^22^ on cancer trial participants, almost half the consent discussions were reported to last 1 hr or longer. It also reported that an adult friend or relative was present for 84% of discussions, whereas in our study, only 7 out of the 20 participants (35%) were accompanied by their partner / relative / friend. Considering that the woman’s partner is expected to play an important role in the decision about trial participation during pregnancy ^34^, this was a low proportion. It can be inferred that most women in this setting consider themselves self-competent and confident enough to decide about their participation on their own.

The Joffe study also ^22^ reported that a nurse was present for 39% of the consent discussions. This proportion was much higher in our study - in 18 of the 20 (90 %) consent encounters observed, which is a very positive finding. It has been suggested that a research nurse plays a crucial role in the consent process in research involving pregnant women. They are in the unique position to share information, reassure, and help in building confidence in the women regarding research participation ^34^. In this study, the research nurses in the study usually made preliminary contact with the potential trial participants and had initial discussions regarding the trial with them before the consent meetings with the investigators. So, presumably quite a few of the participants’ basic queries regarding the study were satisfied by the nurses, and therefore participants required shorter discussions with the investigators and had fewer questions before giving consent. With a larger sample size, it would have been interesting to assess the impact of presence/absence of a research nurse on the understanding of the consent.

Four investigators conducted the consent discussions for the trial. Different investigators have different communication and linguistic skills, training or experience which could lead to variations in the manner and extent of information dissemination to the participants ^31^. This, in turn could lead to differences in comprehension by the participants. Since the sample size was limited and most consents were administered by the principal investigator of EMERGE, the effect of investigators on the understanding scores of participants could not be evaluated. The study population consisted of pregnant women, and their mental and emotional state is generally considered to be different from non-pregnant women ^35^. With that consideration, our findings demonstrate that pregnancy *does not* compromise the women’s ability to comprehend the complex aspects of providing informed consent to any significant extent as the summary knowledge scores obtained were similar to the ones published for trial participants in different settings.

### Motivation to participate

When asked to give the reasons to participate in the trial, participants most commonly cited that they felt they were helping others and contributing to knowledge. Altruism or the ‘feeling good helping mankind’ is a commonly recognised reason for research participation, and so is the feeling of being part of scientific discovery ^24^. Some also felt they would get better care as part of being in the trial which is a valid benefit expected by women from participating in a clinical trial. Understanding participants’ motivation to participate in research can help in devising effective recruitment strategies. It may also help in improved retention rates. It has been suggested that identifying what’s important and acceptable to women is necessary to come up with appropriate and relevant research strategies, individualised to encourage participation ^34^. However, such qualitative research is usually not feasible with large samples, or using time consuming qualitative interviews. So small scale, questionnaire-based studies like this may prove to be a useful alternative.

### Consent decision aid – a supplement for improving informed consent

Providing adequate information to potential trial participants lies at the heart of obtaining informed consent. It is well recognised that in addition to clear and comprehensive communication by the investigators, the use of well-designed tools including written material or audio-visual media may increase the degree of participant’ understanding of the information provided ^32^. Simple interventions, like use of a structured consent template, presence of a professional third party such as a nurse, giving the patient time to consider participation, asking questions, and encouraging careful reading of consent forms, might result in meaningful gains.

‘Decision aids’ have been recognised as a valuable potential means of helping to facilitate a process of decision-making, by providing clear documentation of relevant information related to the study. Very few published studies have explored the use of decision aids in the context of trial participation decisions and have shown some promise ^18, 36^. They have been shown to improve understanding while not increasing anxiety compared to PILs and resulted in low levels of decisional conflict and high levels of satisfaction ^17^. The list of commonly asked questions and their answers derived from the consent meetings, put together in clear and simple language, can be considered a type of decision aid to help the consenting process. It may help allay the commonly expressed concerns or anxieties of the potential participants, thus proving beneficial in improving recruitment rate in the trial, as well as the level of understanding of the participants.

### Limitations

The present study has some inherent limitations as follows:

***Small sample size***. The original proposed sample size of the study could not be achieved. Though the eventually realised sample size of 15 participants (completing study questionnaire) was considerably smaller, previous studies of similar nature have been conducted with highly variable sample sizes which were reported to be 8 – 1789 patients in different settings and using different evaluation methods ^8, 32^. Further research using a larger sample size would definitely add to this body of work, especially since this is such a niche area of pregnancy research. The limited sample size also did not allow us collect sufficient information to assess the effect of level of education, income, age, and other covariates on the level of understanding of the participants. An analysis of the readability of the informed consent form and the information sheet performed with a measure such as Flesch reading ease score could have further added context to the participants’ understanding of informed consent ^37^.

***Bias***. Selection bias ^38^ could have been introduced because a substantial number of potential participants who were initially contacted by the trial research nurse, did not express interest in participation and hence did not engage in further discussions with the trial investigators. Therefore, it is possible that participants whose consent discussions were observed, and who completed the study questionnaire had substantial differences in their attitude or understanding of the informed consent as compared to those who were not interested in participation. Social desirability bias ^39^ could also have played a role in the component of subjective understanding (self-assessment) in the study. As the participants knew that their ability to comprehend information was being assessed, they may have resorted to rating their understanding of the questionnaire statements higher than they actually felt.

***Other limitations***. There was little ethnic diversity in our sample, and we recommend further studies in more varied populations. The study sample was drawn from a single trial on pregnant women from a single site. Thus, it is not recommendable to generalize the findings of this study to other populations or even to other study sites. More research needs to be conducted at multiple sites, and multiple trials on pregnancy to make concrete inferences about consent understanding among pregnant women. Respondents filled out the questionnaire a median of 16 days after consenting to their clinical trials and their understanding of the concepts we measured might have been better at the time of consent than at this later time. Further assessments needed to be made in this regard. The strategy of repeated assessment of understanding over the course of the trial could have been employed if more time was available for the study.

## Conclusion

In conclusion, this study adds to the wealth of research which demonstrates that participants’ understanding for certain components of informed consent is inadequate in a clinical research setting; the unique study population being studied here was pregnant women with GDM. The results reported here are the first attempt to assess the understanding of informed consent and a description of important aspects of the consent process among pregnant women as clinical trial participants, and in research on GDM. It was shown that the grasp of different components of informed consent in pregnant women is similar to other adult populations enrolling in clinical trials. It was interesting to see that the women tend to consistently overestimate their own understanding of informed consent when asked to subjectively assess it.

The study also highlights that the concerns of pregnant women regarding the safety of their developing foetus are the main parameter of their decision to consent. The positive attitude of the participating women was a reassuring aspect to observe. A simple consent decision aid containing a list of frequently asked questions and their answers was developed as a part of this study which may help in improving their understanding of the important aspects of the trial. Ongoing and future research must be encouraged to determine the best way to conduct the informed consent process, and to ensure that research participants are making truly informed decisions to take part in clinical research studies.

## Data availability

Data underlying this study is available from Open Science Framework (OSF): Project - Assessment of the Understanding of Informed Consent including Participants’ Experiences, and Generation of a Supplemental Consent Decision Aid for Gestational Diabetes Mellitus (GDM) Research. http://doi.org/10.17605/OSF.IO/7ABPR, Held under license: CC0 1.0 Universal

## Supplementary material

Supplementary File 1 – Study Questionnaire

Supplementary File 2 - Participant Information Sheet

Supplementary File 3 - Consent decision aid – EMERGE
