# Supplementary material for: Assessment of the understanding of informed consent including participants’ experiences, and generation of a supplemental consent decision aid for Gestational Diabetes Mellitus (GDM) research
Source: HRB Open Res. 2018 Mar 29;1:12. [Version 1] doi: 10.12688/hrbopenres.12811.1 (PMC6973531; doi:10.12688/hrbopenres.12811.1)
Supplement: Supplementary file 1 [file hrbopenres-1-13871-s0000.tgz › 22c79af2-a482-4bd4-87a0-a2dbe2926ad4.docx]

**STUDY QUESTIONNAIRE**

**Study title: Assessment of the informed consent process including participants’ experiences, and generation of a supplemental consent decision aid for gestational diabetes mellitus (GDM) research.**

*(Participant Number: )*

**Quality of Informed Consent (QuIC), Part A**

**INSTRUCTIONS:**

Below you will find several statements about clinical trials (otherwise known as research studies). Thinking about your clinical trial, please read each statement carefully. Then tell us whether you agree with the statement, you disagree with the statement, or you are unsure about the statement by circling the appropriate response. Please respond to each statement as best as you can. We are interested in your opinion.

| A1. When I signed the consent form for my current therapy, I knew that I was going to participate in a clinical trial. | Agree | Unsure | Disagree |
| --- | --- | --- | --- |
| A2. The main reason clinical trials are done is to improve the treatment of future patients. | Agree | Unsure | Disagree |
| A3. I have been informed how long my participation in the clinical trial is likely to last. | Agree | Unsure | Disagree |
| A4. All the treatments and procedures in my clinical trial are standard for my type of condition. | Agree | Unsure | Disagree |
| A5. In my clinical trial, one of the researchers’ main purposes is to compare the effects (good or bad) of two or more different ways of treating patients with my type of condition, in order to see which is better. | Agree | Unsure | Disagree |
| A6. The treatment being researched in my clinical trial has been proven to be best for my type of condition. | Agree | Unsure | Disagree |
| A7. After I agreed to participate in my clinical trial, my treatment was chosen randomly (by chance) from two or more possibilities. | Agree | Unsure | Disagree |
| A8. Compared with standard treatments for my type of condition, my clinical trial does not carry any additional risks or discomforts. | Agree | Unsure | Disagree |
| A9. There may not be direct medical benefit to me from my participation in this clinical trial. | Agree | Unsure | Disagree |
| A10. By participating in this clinical trial, I am helping researchers learn information that may benefit future patients. | Agree | Unsure | Disagree |
| A11. Because I am participating in a clinical trial, it is possible that the study sponsor, various government agencies, or others who are not directly involved in any care could review my medical records. | Agree | Unsure | Disagree |
| A12. My doctors did not offer me any alternatives besides treatment in this clinical trial. | Agree | Unsure | Disagree |
| A13. The consent form I signed describes who will pay for treatment if I am injured or become ill as a result of participation in this trial. | Agree | Unsure | Disagree |
| A14. The consent form I signed lists the name of the person (or persons) whom I should contact if I have any questions or concerns about this clinical trial. | Agree | Unsure | Disagree |
| A15. If I had not wanted to participate in this clinical trial, I could have declined to sign the consent form. | Agree | Unsure | Disagree |
| A16. I will have to remain in the clinical trial even if I decide someday that I want to withdraw. | Agree | Unsure | Disagree |

**Quality of Informed Consent (QuIC), Part B**

INSTRUCTIONS: When you signed the consent form to participate in your clinical trial, how well did you understand the following aspects of your clinical trial? If you didn’t understand the item at all, please circle 1. If you understood it very well, please circle 5. If you understood it somewhat, please circle a number between 1 and 5.

|  | I didn’t I understood  understand this very  this at all well | | | | |
| --- | --- | --- | --- | --- | --- |
|  |  |  |  |  |  |
| B1. The fact that your treatment involves research | 1 | 2 | 3 | 4 | 5 |
| B2. What the researchers are trying to find out in the clinical trial | 1 | 2 | 3 | 4 | 5 |
| B3. How long you will be in the clinical trial | 1 | 2 | 3 | 4 | 5 |
| B4. The treatments and procedures you will undergo | 1 | 2 | 3 | 4 | 5 |
| B5. Which of these treatments and procedures are experimental | 1 | 2 | 3 | 4 | 5 |
| B6. The possible risks and discomforts of participating in the clinical trial | 1 | 2 | 3 | 4 | 5 |
| B7. The possible benefits to you of participating in the clinical trial | 1 | 2 | 3 | 4 | 5 |
| B8. How your participation in this clinical trial may benefit future patients | 1 | 2 | 3 | 4 | 5 |
| B9. The alternatives to participation in the clinical trial | 1 | 2 | 3 | 4 | 5 |
| B10. The effect of the clinical trial on the confidentiality of your medical records | 1 | 2 | 3 | 4 | 5 |
| B11. Who will pay for treatment if you are injured or become ill because of participation in this clinical trial | 1 | 2 | 3 | 4 | 5 |
| B12. Whom you should contact if you have questions or concerns about the clinical trial | 1 | 2 | 3 | 4 | 5 |
| B13. The fact that participation in the clinical trial is voluntary | 1 | 2 | 3 | 4 | 5 |
| B14. Overall, how well did you understand your clinical trial when you signed the consent form | 1 | 2 | 3 | 4 | 5 |

***Why did you agree to participate in the trial?***

*Please tick as many as appropriate*

1. *I thought the trial offered the best treatment available*
2. *I believe results from the trial could benefit other patients in the future*
3. *I want to contribute to scientific research*
4. *I believe I will be monitored more closely as part of this trial*
5. *I believe the quality of care I receive will be better as part of this trial*
6. *My family were keen for me to participate*
7. *I trusted the doctor treating me*
8. *I think my cancer will get worse unless I take part in the trial*
9. *Other (please give details below)*

***________________________________________________________________***

***How would you describe your overall satisfaction with the informed consent process for the trial?***

*(Please tick)*

*Very satisfactory / Satisfactory / Neutral / Unsatisfactory / Very unsatisfactory*

***Demographic details:***

**Age:**

**Parity**:

**Ethnicity:** (Please tick)

Caucasian / White

African / Afro American / Black

Asian

Hispanic / Latino

Others

**Level of education:** (Please tick)

Primary

Junior or Inter certificate

Leaving Certificate

Third level – Bachelor’s

Third level – Master’s or higher
