# Supplementary material for: Assessment of the understanding of informed consent including participants’ experiences, and generation of a supplemental consent decision aid for Gestational Diabetes Mellitus (GDM) research
Source: HRB Open Res. 2018 Mar 29;1:12. [Version 1] doi: 10.12688/hrbopenres.12811.1 (PMC6973531; doi:10.12688/hrbopenres.12811.1)
Supplement: Supplementary file 2 [file hrbopenres-1-13871-s0001.tgz › 9dce059f-566a-46d4-94be-a89c42368807.docx]

**Participant Information Sheet**

**Study title: Assessment of the informed consent process including participants’ experiences, and generation of a supplemental consent decision aid for gestational diabetes mellitus (GDM) research.**

**Objective:**

This study is being conducted to assess the quality of the informed consent process in the EMERGE clinical trial and the patients’ experience of the process. The study also aims to generate a simple written supplemental aid containing common questions / concerns raised by patients with answers to help future patients decide about participation in GDM trials.

**Introduction:**

You are being asked to take part in this study because you have consented to participate in the EMERGE clinical trial. If you agree to take part, we will ask you to sign a Consent Form. Taking part in this study is voluntary. If you don’t want to take part, you don’t have to give a reason, and your decision will not affect the care you receive. Even if you change your mind later, you can pull out of the study at any time.

We will go through this information with you and answer any questions you may have. If there is anything that you are not clear about, we will be happy to explain it to you. Please take as much time as you need to read it. You should only consent to participate in this research study when you feel that you understand what is being asked of you, and you have had enough time to think.

Thank you for reading this.

**Purpose of the study:**

Our study is looking at the quality of the informed consent that is taken for your participation in the EMERGE clinical trial, which aims to evaluate the use of metformin in pregnant women diagnosed with GDM. We want to assess your experiences and opinions about the informed consent process including the common questions or concerns raised by you during the process. Informed consent is a fundamental ethical requirement for research. We want to learn from participants’ feedback how we can improve this process.

We will measure the quality of the consent process and record your experiences through a standardized questionnaire. We will observe the interaction between participants and the investigator/s in the consent process to find out the common questions / concerns raised.

**Taking part – what it involves:**

***Do I have to take part?***

Participation in the study is voluntary. A decision to withdraw at any time, or a decision not to take part, will not affect your rights or medical care in any way.

***What is expected of me if I take part?***

If you decide to take part, you will be given a standard questionnaire to fill out on one of your first scheduled visits for the EMERGE trial. The study questionnaire will contain 32 simple statements / questions regarding the informed consent process of EMERGE clinical trial, and will take an average of 10 mins to fill out. You can respond by circling the most appropriate option among the answers provided. The questionnaire will be completed by you in the presence of the study investigator, who can help you to understand any statement / question that you may not understand. Your demographic details like age, race, parity, and level of education will be collected as part of the questionnaire.

***How long will my part in the study last?***

Your participation in the study would involve answering the study questionnaire once during one of the scheduled follow up visits (preferably the first visit at 2 weeks) for the EMERGE clinical trial.

***What do I have to do?***

Other than allowing the observation of your interactions during the consent meeting/s and answering the study questionnaire, you do not have to do anything else for this study.

***What are the possible benefits in taking part?***

By taking part you would be contributing to improving the quality of the informed consent process for future participants of research.

***What are the possible disadvantages and risks of taking part?***

There are no foreseeable disadvantages or risks.

***What happens if I change my mind during the study?***

You are entitled to change your mind at any time during the study without any disadvantage or penalty.

***Whom do I contact for more information or if I have further questions or concerns?***

If you want any information or have questions or concerns, you can contact Dr. Shubham Atal, who is the investigator for this study at: shubham.atal@gmail.com, Mobile: +353 899878241

If you wish to contact someone independent and in confidence, you may contact the office of the Chairperson of the Galway University Hospitals Research Ethics Committee, Tel: 091 775022.

**Confidentiality:**

All information that is collected about you during the course of the research will be kept strictly confidential and will not be shared with anyone else. Results from the study will be reported as group data and will not identify you in any way.

Dr. Shubham Atal Prof. Fidelma Dunne

MSc candidate (Clinical research) School of Medicine,

HRB-CRF Galway, NUI Galway, Ireland NUI Galway, Ireland

s.atal1@nuigalway.ie, +353899878241, fidelma.dunne@nuigalway.ie

**Version number: 3.0 Dated: 29^th^ March 2017**

**CONSENT FORM**

**Title of Project: Assessment of the informed consent process including participants’ experiences, and generation of a supplemental consent decision aid for gestational diabetes mellitus (GDM) research.**

Name of Investigator: Dr. Shubham Atal

Name of Supervisor: Prof. Fidelma Dunne

**Please initial box**

I. I confirm that I have read the information sheet dated 29/03/2017 (version 3.0).

2. I am satisfied that I understand the information provided.

3. I understand that my participation is voluntary.

4. I agree to take part in the above study.

Name of Participant: Date: Signature

Name of Investigator: Date: Signature

**Version number: 3.0 Dated: 29^th^ March 2017**

**(1 copy of consent form to be provided to participant; 1 copy to be kept by investigator)**
