# Supplementary material for: Assessment of the understanding of informed consent including participants’ experiences, and generation of a supplemental consent decision aid for Gestational Diabetes Mellitus (GDM) research
Source: HRB Open Res. 2018 Mar 29;1:12. [Version 1] doi: 10.12688/hrbopenres.12811.1 (PMC6973531; doi:10.12688/hrbopenres.12811.1)
Supplement: Supplementary file 3 [file hrbopenres-1-13871-s0002.tgz › 1e686427-961b-47a1-849c-df224a908e90.docx]

**CONSENT DECISION AID - EMERGE**

**(Common questions / concerns of EMERGE participants)**

1. **Is metformin safe for the baby? What can be the short term and long term effects on the baby?**

There is a strong body of evidence demonstrating safety of metformin when used for Gestational Diabetes Mellitus (GDM) and other conditions like Polycystic Ovarian Syndrome (PCOS) in pregnant and non-pregnant women. Combined data on more than 1500 participants with gestational diabetes from multiple studies has shown no increase in birth defects or adverse outcomes in babies with metformin, and found it to be better than insulin in terms of reducing excess weight gain in mothers and preventing excessively large babies.

Follow up studies up to 18 – 24 months for long term effects of metformin on babies have shown normal weight, height and motor or social skills in the babies. There have been other studies too, in up to 100 – 1000 women, which have shown better pregnancy outcomes for both mother and baby with use of metformin for GDM.

1. **Has metformin been used previously? What are the other uses of metformin in women?**

Although not used in Ireland currently, metformin has been in use for GDM in other European countries including UK, as well as Australia, New Zealand, South Africa and Canada.

Metformin has been extensively used for treatment of type 2 diabetes mellitus and PCOS – polycystic ovarian syndrome which is a common cause of infertility in women of reproductive age group.

1. **How many patients will be recruited in the trial?**

A total of 550 patients are targeted to be recruited in the trial at the two hospitals in Ireland - University Hospital Galway and University Hospital Cork.

1. **What are the benefits of starting metformin early in GDM? Does metformin reduce risk of diabetes after pregnancy?**

Metformin has been shown to be safe in pregnancy (for mother and baby), when it is introduced after failure of diet and exercise. Studies have found metformin to be slightly better than insulin with additional advantages like ‘needle less treatment’, prevention of excessive weight gain in mother and baby, reduced risk of hypoglycaemia (low blood sugar), reduced risk of diabetes in the future for the mother, pregnancy induced hypertension, preeclampsia and caesarean delivery. Metformin may also reduce future risk of cardiovascular events and cancer.

So, it is reasonable to expect that the impact on control of blood sugar and additional benefits of metformin for mother and baby would be greater if metformin is started early (at the time of diagnosis) along with the usual care i.e. diet and exercise interventions.

1. **What are the common adverse effects of metformin? Does it cause hypoglycaemia?**

The most commonly reported side effects of metformin are nausea and vomiting, diarrhoea, and flatulence. Other very rare side effects of metformin include lactic acidosis (increased lactate in the blood) and decreased vitamin B12 levels usually associated with long term use.

Metformin works to reduce blood glucose in a way that it is not expected to cause hypoglycaemia usually.

1. **Will I be on metformin or placebo?**

Treatment for each participant will be decided in this study at random (by chance alone) to receive either metformin or the ‘dummy’ treatment called placebo; so, every patient will have an equal chance of receiving metformin or placebo.

1. **What is the time required to be spent in hospital for the study during pregnancy– are any additional visits required? How long will the visits be?**

As a participant in the study, you will be expected to attend for follow up visits to the study site every 2 weeks till the birth of your baby which is also usually the frequency of antenatal visits. These follow up visits will occur on the same day as your routine antenatal visits, thus avoiding extra visits. Aside from the time for antenatal visits, the follow up visits would require 15 – 30 mins depending on visit requirements.

After delivery, the after birth postnatal visit will be conducted in the ward itself, a telephone visit will be conducted at 4 weeks, and you will be asked to attend for a final study visit 12 weeks after delivery.

1. **What is bio banking?**

Bio banking is the storing of biological samples like blood, placental and umbilical cord in this study for use in related future research.

1. **How long will the trial take to complete?**

Each participant will be in the trial from the time of diagnosis of GDM and entry into the study until the last follow up visit which is at 12 weeks after delivery.

Overall, the trial is expected to recruit 550 patients across the two sites in 2 – 3 years.

1. **Is self-monitoring of blood being done to decide about initiation of insulin? What should be the timing of self-monitoring? Will I need to write down monitoring results?**

During the study, you may or may not require insulin depending on the control of your blood glucose. Home blood glucose monitoring is usually recommended as part of usual care and you will be expected to do blood glucose measurements 7 times in a day – before and 1 hour after every meal and before bed with the help of the study glucometer provided, so that trends in blood glucose levels are monitored.

The blood glucose values will be downloaded from your glucometer at every study visit. Additionally, you are also expected to write down the values in your own notes.

1. **Will visits be needed after delivery?**

After delivery, the post-natal visit will be conducted in the ward itself, a telephone visit will be conducted at 4 weeks, and you will be expected to come only for a final study visit 12 weeks after delivery.
