# Supplementary material for: Assessment of the understanding of informed consent including participants’ experiences, and generation of a supplemental consent decision aid for Gestational Diabetes Mellitus (GDM) research
Source: HRB Open Res. 2018 Mar 29;1:12. [Version 1] doi: 10.12688/hrbopenres.12811.1 (PMC6973531; doi:10.12688/hrbopenres.12811.1)
Supplement: Peer review history

# Peer review: Reviewer response for version 1

**DOI:** 10.21956/hrbopenres.13871.r26270

**Published:** 2018-06-25

**Authors:** Sasitorn Phumdoung

**Affiliations:** 1 Faculty of Nursing, Prince of Songkla University, Songkhla, Thailand

Methods: 

**The strong point**

 The  research design and data collection are appropriate.

**The weakness**

 The authors did not report on validity and reliability of their questionnaires.

 The author did not provide sample size calculation or the sufficient of samples.

 In resulting part Table 1 should be shown using Figure.

 Also the report part should provide information more on the total scores.

**Discussion part**

 The authors should inform all of the topics needing to report at the beginning of the discussion section to make more understandable to the readers.

I confirm that I have read this submission and believe that I have an appropriate level of expertise to confirm that it is of an acceptable scientific standard.

# Peer review: Reviewer response for version 1

**DOI:** 10.21956/hrbopenres.13871.r26272

**Published:** 2018-06-25

**Authors:** John F. Forbes

**Affiliations:** 1 Health Research Institute (HRI), University of Limerick, Limerick, Ireland

This is a well conducted, largely exploratory, analysis of how well participants in a randomised controlled trial (RCT) understand important aspects of informed consent. It is a relatively small scale study embedded within a larger trial. Selection bias can be problematic in studies within a trial and this study is no exception as it isn't clear how representative this sub-set of participants is compared to women who were recruited to the EMERGE trial. 

 Using validated instruments information is collected which enables analysis of both "subjective" and "objective" understanding or awareness of selected aspects of the consent process and its implications. It isn't clear whether these instruments are adequate, or could be improved, when applied to choices made by women with respect to their own health and well being as well as that of their foetus. There may be similar issues when informed consent is sought from parents' for their children who may be recruited to experimental studies.  

 The gaps in understanding demonstrated by this study deserve further attention. Informed consent must be a iterative process whereby initial understanding is enhanced and progressed. Another issue is how participants are recruited to experimental studies and the extent to which potential recruits are given useful information before they are invited to consent.

I confirm that I have read this submission and believe that I have an appropriate level of expertise to confirm that it is of an acceptable scientific standard.

# Peer review: Reviewer response for version 1

**DOI:** 10.21956/hrbopenres.13871.r26144

**Published:** 2018-05-23

**Authors:** Eleanor M. Scott

**Affiliations:** 1 Division of Epidemiology and Biostatistics, Leeds Institute of Cardiovascular and Metabolic Medicine, University of Leeds, Leeds, UK

Clinical trials in pregnant women are uniquely difficult because of the implications for the fetus. It is therefore imperative that women participating in such trials fully understand the risks/benefits/safeguards of taking part, as exemplified by the informed consent process. Whilst participant understanding of the informed consent process has been explored in other 'non-pregnant' situations, this is the first study to evaluate participant understanding in the context of a clinical trial in pregnancy. Using validated questionnaires the study found that whilst there was good overall understanding of the process of informed consent, there was a mismatch between objective and subjective understanding of three specific areas: non-standard nature of treatment, additional risks/discomforts and compensation. The authors conclude that development and use of a decision aid may help a participant to understand these areas more clearly.

 The work is well written and appropriately cites current literature. The study design is sound, although the number of participants is small. The questionnaire to assess understanding of the informed consent process was administered 2-4 weeks after the actual time of informed consent. This raises the possibility of recall bias affecting the results. It is unclear to me why this wasn't administered immediately after the informed consent process, although I can understand that this delay may have been chosen to avoid overburdening the women at a critical point in the clinical trial process. The methods and data analysis are clear, as is the interpretation of the findings. Although the source data are available, the link wouldn't work when I tried it. The discussion and conclusions are sound.

 Overall this study gives some very useful insights for researchers doing clinical trials in pregnancy, and how to improve the informed consent process.

I confirm that I have read this submission and believe that I have an appropriate level of expertise to confirm that it is of an acceptable scientific standard.
